# Pneumatosis cystoides intestinalis: A complicated case presentation

**DOI:** 10.1016/j.amsu.2022.104514

**Published:** 2022-09-08

**Authors:** Rudo Pswarayi, Claire Joy Sanders

**Affiliations:** a Department of General Surgery, Chris Hani Baragwanath Academic Hospital, Faculty of Health Sciences, University of the Witwatersrand, Johannesburg, South Africa; b Department of General Surgery, Helen Joseph Hospital, Faculty of Health Sciences, University of the Witwatersrand, Johannesburg, South Africa

**Keywords:** Bowel obstruction, Embryonic duplication, Gastric volvulus, Gastrointestinal cysts, Gastric outlet obstruction

## Abstract

**Introduction:**

Pneumatosis Cystoides Intestinalis (PCI) is a fairly rare condition that is, however, becoming of increasing incidence. It is difficult to ascertain as many patients are asymptomatic, but, in adults, if it does present it usually presents in the fifty-to-eighty years age groups.

**Importance:**

It may be primary (15% - idiopathic and benign condition) or secondary (85% - usually in neonates and due to necrotising enterocolitis) but the aetiology is unclear, with some theories that have been put forward. Many consider its pathogenesis to be due to increased intra-luminal intestinal pressure and, therefore, build-up of gas from commensal gut bacteria. Imaging and histology are important in diagnosis.

**Case presentation:**

This case presentation explores the complicated presentation of a patient with PCI and his management, thereof, in order to review the appropriate investigations and the subsequent options of management.

**Conclusion:**

Because it is a poorly understood condition with varying presentations, it is often misdiagnosed. The diagnosis is critical to allow the correct and most appropriate management to be carried out: non-surgical supportive care versus surgical intervention with resection and stoma or anastomosis.

## Highlights

Pneumatosis cystoides intestinalis: presentation and management of a complicated case.

● Pneumatosis Cystoides Intestinalis (PCI) is a fairly rare condition and is difficult to diagnose as many patients are asymptomatic.
● Imaging and histology are key the in diagnosis and subsequent non-surgical versus surgical management.
● This case presentation explores the complicated presentation of a patient with PCI and his management, along with some associated concurrent diagnoses.

## 1 Introduction

This case report has been reported in line with the SCARE Criteria [Agha RA, Franchi T, Sohrabi C, Mathew G, for the SCARE Group. The SCARE 2020 Guideline: Updating Consensus Surgical CAse REport (SCARE) Guidelines, International Journal of Surgery 2020; 84:226–230] [7].

Pneumatosis Cystoides Intestinalis (PCI) is a very uncommon condition that may be associated with different diseases. It is characterized by the presence of multiple gaseous cysts in the intestinal wall that contain a mixture of carbon dioxide, nitrogen, and hydrogen. Commonly, the cysts are located beneath the serosa and mucosa of the intestinal wall with majority being in the ileum, followed by the colon, and at times (but rarely) in both small bowel and colon [1]. The peak age of onset is 45years with a higher predilection for males (male: female = 2.4:1).

The aetiology of PCI is not known, it may be primary or secondary, but has been associated with gastrointestinal surgeries, Chronic Obstructive Pulmonary Disease (COPD), laxative use, connective tissue disorders, malnutrition, alpha-glucosidase inhibitor, and colonoscopies [2]. Primary PCI involves the development of multiple thin-walled cysts that occur in intestinal submucosal and serosal layers. Some theories put forward with regards to pathophysiology include the pulmonary, bacterial, and mechanical theories. 1. Pulmonary: patients with asthma and chronic bronchitis may have alveoli that rupture, gas travels through the mediastinum into the retroperitoneum which then enters the perivascular spaces in the intestinal wall [2]. 2. Bacterial: Clostridia and Escherichia Coli localize in the submucosa and produce gas during fermentation, and this is retained in the submucosa and lymphatic vessels. This may explain why administration of Metronidazole may result in resolution of the pneumatosis [2]. 3. Mechanical: after trauma, surgery, colonoscopy or bowel obstruction, bowel gas is translocated through a mucosal defect into lymphatic vessels, and it is propagated by peristalsis [2].

Clinical examination may include symptoms of abdominal pain, nausea and vomiting, diarrhoea, haematochezia, or may present complicated with pneumoperitoneum, volvulus, intestinal obstruction, and ischaemia [3]. Non-surgical resolution of disease has been reported in up to 70%; patients may improve with fasting, fluid replacement, cessation of alpha-glucosidase inhibitors, oxygen/hyperbaric oxygen administration, administration of metronidazole or quinolones, and endoscopic therapies (Fine needle aspiration, high-frequency endoscopic resection) [3].

In this case presentation, the complex findings of multiple visceral cystic lesions, a gastric volvulus and gastric outlet obstruction present a challenge to the surgical approach and management of such a patient.

## 2 Case presentation

### 2.1 Presentation

A 39-year-old black African male presented to a secondary facility emergency department complaining of a two-week history of progressive abdominal distension with one day of associated severe abdominal pain and post-prandial vomiting. No history of loss of weight, no past surgical history, and no medical history or chronic medication use. There was no family history of note, and the patient had no history of smoking or alcohol intake.

### 2.2 Clinical examination

General examination: underweight patient, no palpable lymph nodes Heart Rate (HR) = 114 beats per minute; Blood Pressure (BP) = 103/59 mmHg, Temperature = 36.4° Celsius, Respiratory Rate (RR) = 27 breaths per minute.

Abdominal examination: massively distended, tense, tympanic abdomen, peritonitic. Succussion splash present. Per rectum examination: soft brown stool with a normal prostate. Cardiovascular and respiratory examinations were normal.

### 2.3 Pre-operative management

Two large bore intravenous (IV) lines were inserted and 1 L crystalloid fluid bolus given with subsequent crystalloid infusion rate of 125ml/hr IVI. Urine catheter inserted drained 600ml clear urine, nasogastric tube (NGT) inserted and 3.5 L of gastric material drained with some relief of abdominal distension and pain (Table 1).

**Table 1:** Initial arterial blood gas and blood results.

Arterial Blood Gas	Value
pH	7.234
pCO_2_	26
pO_2_	89
Saturation	99
Lactate	5.2
Base Excess	−7.6
Bicarbonate	16.8
Chloride	81
Sodium	126
Potassium	2.3
Haemoglobin	12.3

*Blood Results*							
White Cell Count (x10^9^/L)	Haemoglobin (g/dL)	Mean Corpuscular Volume (fL)	Platelet count (x10^9^/L)	Urea (mmol/L)	Creatinine (micromol/L)	C-Reactive Protein (mg/L)	Amylase (U/L)
9.22	12.4	82.4	273	10.9	101	60	152

**Fig. 1:**
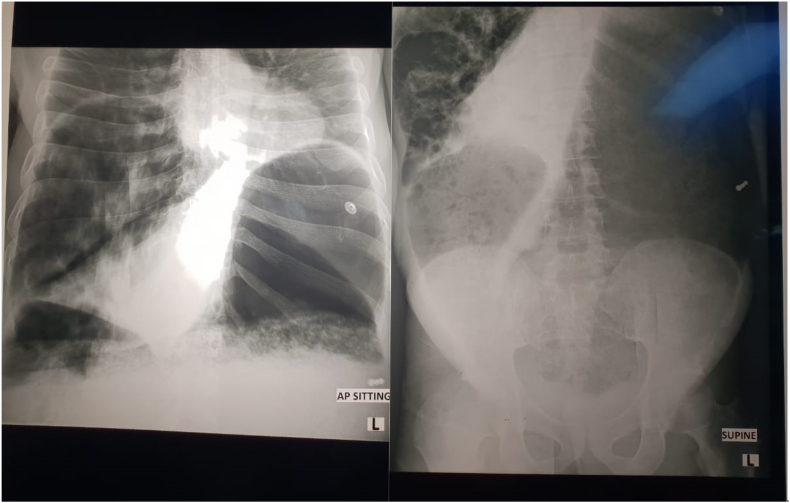
Abdominal X-Ray - Distended pelvic stomach and right upper quadrant cystic appearance.

Chest X-ray: bilaterally elevated diaphragms with small but clear but reduced lung fields and no free air under the diaphragm, Abdominal X-Ray (Fig. 1): large right upper quadrant cystic lesion, large, distended gastric bubble with possible colon distension, no air in the rectum. COVID swab: negative. Patient was booked for emergency laparotomy for peritonitic bowel obstruction with possible bowel perforation.

### 2.4 Intra-operative management

**Fig. 2:**
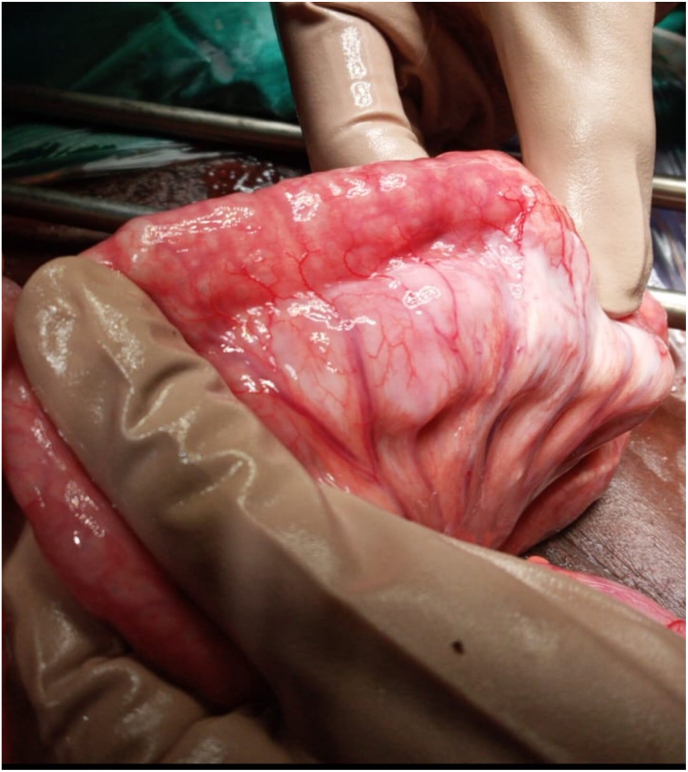
Ante-mesenteric cystic duplication parallel to jejunum 110cm from DJ Flexure extending to distal ileum.
5) Massive supra-hepatic cystic mass of 15cmx12cm

Theatre:

1) Pneumoperitoneum noticed on entering the peritoneum
2) Massively dilated pelvic stomach with Organo-axial gastric volvulus – viable.
3) Narrow pylorus (<0.5cm)
4) Multiple extensive cystic mesenteric-para -jejunal-ileal-colonic duplication cysts starting 110cm from Duodenal-Jejunal (DJ) Flexure to proximal ascending colon (Fig. 2).

### 2.5 Procedures undertaken

- Detorsion of gastric volvulus – viable distal oesophagus and stomach – gastropexy performed along the anterior fundus.
- All cystic-duplication intestinal cysts left intact: true intestinal lumen viable and peristalsis present throughout.
- Supra-hepatic cystic lesion left in situ with pencil drain placed inferiorly

### 2.6 Post-operative management

**Fig. 3:**
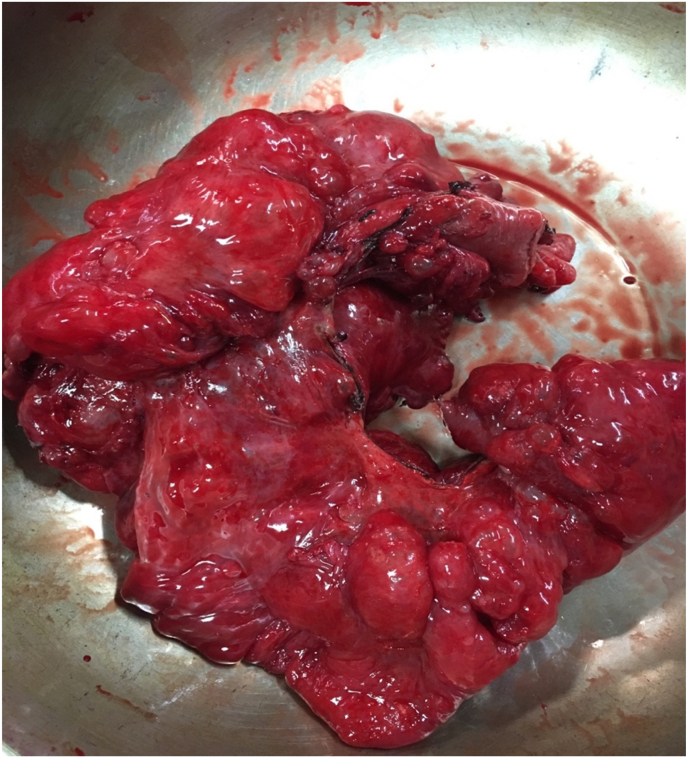
Resection of 80cm of jejunum and ileum containing cystic lesions.

Patient was extubated on table and transferred to the ward with NGT on free drainage and IVF support. Enhanced Recovery After Surgery (ERAS) protocol was followed and feeds were progressed from clear fluids to soft diet withing 48 hours post operatively. Patient noted to have post-prandial vomiting within 1–2 hours after eating on day six post-surgery, subsequently oral feeds were stopped and patient kept nil per os on IVF support. Booked for a Gastroscopy and Barium Meal and follow-through. Gastroscopy: large amounts of gastric content – unable to view stomach. Gastric lavage was carried out and repeat Gastroscopy after 48 hours showed: Gastric Outlet Obstruction, pin-hole pylorus visualized and 6× biopsies taken. Barium meal and follow-through on day ten post-surgery demonstrated a large pelvic stomach with delayed transit of contrast into small bowel and subsequent large bowel. At this point the patient tolerating clear sips per os and supported with Total Parenteral Nutrition (TPN). Abdomen otherwise soft and not tender, minimal distention. *Histology Report of Biopsies:* No Helicobacter Pylori organisms noted; gastric mucosa shows widespread chronic gastritis, no active inflammation, no evidence of lymphoid follicle formation, no evidence of dysplasia or invasive malignancy. Day 19 post-surgery pyloric stenting was attempted but failed. Elective surgery planned involving a distal gastrectomy with Bill Roth 2 (Gastrojejunostomy) procedure, Jejunal and ileum cyst-lesion resection (Fig. 3) with small bowel-small primary anastomosis, resection of supra-hepatic cystic lesion – this was performed Day 23 post initial laparotomy. Thereafter, the patient progressed with feeds well to a full diet. Histology of the specimens showed: All gastric tissue margins do not demonstrate invasive malignancy, widespread chronic gastritis noted in mucosa, no active inflammation, no H. pylori noted. Jejunum-ileum tissue: morphological features of pneumatosis cystoides intestinalis, no neoplastic infiltrate. Liver cyst resection: morphological features of pneumatosis cystoides intestinalis, nil neoplasia. The patient was discharged thirty-one days after his presentation to the emergency department.

## 3 Discussion

In the above patient case, no incitive cause was identified to result in the PCI, however, operative findings and histology confirmed the diagnosis.

PCI is a disease that at times may be confused with intestinal polyps, inflammatory bowel disease, or malignancy. Plain abdominal Xray (pneumoperitoneum, linear/curvilinear gas collection in intestinal wall), Computed Tomography (CT) (Most sensitive showing mesenteric stranding, bowel wall thickening, and ascites), colonoscopy (polypoid protruding lesions) and histology assist in confirming this diagnosis [4]. The histology is characterized by submucosal and subserosal empty spaces that are lined by multinucleated histiocytes or giant cells. The mucosa may also contain cryptitis, crypt abscesses or granulomas; and it may also represent lipomatosis [5]. PCI has been associated with Crohn's Disease and they may both co-exist.

Majority of PCI lesions are in fact found in the colon (approximately 46%), followed by the small bowel in roughly 27%, and then stomach in less than 5%. It may occur in both large and small bowel in 7% of patients [6]. The presentation of PCI includes abdominal pain, diarrhoea, nausea and vomiting, and haematochezia. In less than 3% it may present as bowel obstruction, intestinal volvulus, pneumoperitoneum or bowel ischaemia [6].

Surgical intervention is not necessarily indicated if no complication exists on presentation, and the condition may be managed with oxygen administration, antibiotic treatment if sepsis is proven, cessation of any causative agents or medication, and a trial of nil per os with intravenous fluid support. Surgical intervention depends on localization of the cystic lesions and clinical signs of complication such as bowel obstruction, septicaemia, perforation, or peritonitic abdomen with associated hypotension and/or acute kidney injury.

In the above case study, the patient presented with an acute peritonitic abdomen and imminent signs of hypotension and acute kidney injury. The gastric volvulus was managed in the initial laparotomy with detorsion and gastric pexy, however, as there was no further mechanical small bowel or large bowel obstruction, the hepatic and small bowel cystic lesions were managed conservatively. Supportive management was ensued here forth, but, the further development of gastric outlet obstruction from the thickened pylorus necessitated a relook laparotomy involving a distal gastrectomy with a gastrojejunostomy and resection of the hepatic cyst.

## 4 Conclusion

PCI is not a common disease but, however, its increasing in incidence makes it important to be aware of as a differential diagnosis. Imaging and histology are of utmost importance in its diagnosis. Conservative non-operative management may be carried out in stable and uncomplicated patients, which involves oxygen administration, antibiotic treatment, maintaining a patient nil-per-os, cessation of alpha-glucosidase inhibitors, and intravenous fluid support, with an optimistic prognosis and outcome in over 70% of case. In the limited cases endoscopic treatment with fine needle aspiration or resection may be done if disease is confined. In complicated cases of obstruction, septicaemia, or perforation surgery may be undertaken with resection of the affected portion of viscera with resultant anastomosis or stoma depending on the case. As demonstrated by this case, both non-operative and eventual operative managements of PCI were undertaken demonstrating the complexity of its management.

## Ethical approval

University of Witwatersrand Human Research Ethics Committee (HREC) approved the case report. Ethics approval was approved by the Ethics Committee.

## Ethics

Written informed consent was obtained from the patient for publication of this case report and any accompanying images. A copy of the written consent is available for review by the Editor-in-Chief of this Journal.

## Funding

The author(s) received no financial support for the research, authorship, and/or publication of this article.

## Author contribution

All Authors contributed equally to this case report.

## Consent

Informed Consent given.

“Written informed consent was obtained from the patient for publication of this case report and accompanying images. A copy of the written consent is available for review by the Editor-in-Chief of this journal on request”.

## Registration of research studies

1. Name of the registry:

National Health Research Database.

2. Unique Identifying number or registration ID:

GP 202 2070 40.

3. Hyperlink to your specific registration (must be publicly accessible and will be checked):

https://nhrd.health.gov.za.

## Guarantor

Rudo Pswarayi.

## Availability of supporting data

Not applicable.

## Annals of medicine and surgery

The following information is required for submission. Please note that failure to respond to these questions/statements will mean your submission will be returned. If you have nothing to declare in any of these categories then this should be stated.

## Declaration of competing interest

The author(s) declared no potential conflicts of interest with respect to the research, authorship, and/or publication of this article.

## Abbreviations

Pneumatosis Cystoides Intestinalis: (PCI)
Chronic Obstructive Pulmonary Disease: (COPD)
Heart Rate: (HR)
Blood Pressure: (BP)
Respiratory Rate: (RR)
Intravenous: (IV)
Nasogastric tube: (NGT)
Duodenal-Jejunal: (DJ)
Enhanced Recovery After Surgery: (ERAS)
Total Parenteral Nutrition: (TPN)
Computed Tomography: (CT)
